# Dentoalveolar, skeletal, pharyngeal airway, cervical posture, hyoid bone position, and soft palate changes with Myobrace and Twin-block: a retrospective study

**DOI:** 10.1186/s12903-023-02773-x

**Published:** 2023-01-30

**Authors:** Zeynep Çoban Büyükbayraktar, Hasan Camcı

**Affiliations:** 1 Department of Orthodontics, Faculty of Dentistry, Sivas Cumhuriyet University, Sivas, Turkey; 2 Department of Orthodontics, Afyonkarahisar Health Science University, Afyonkarahisar, Turkey

**Keywords:** Functional orthodontic appliances, Myobrace, Twin-block, Class II div 1 malocclusion

## Abstract

**Background:**

The primary aim of this study was to evaluate the dentoalveolar, skeletal, pharyngeal airway, cervical posture, hyoid bone position, and soft palate effects of the Myobrace and Twin-block appliances. The second was to compare them in terms of ease of use by assessing the factors that may influence patient compliance.

**Methods:**

The study included thirty-six Class II division 1 patients (19 females, 17 males; mean age, 12.14 ± 1.23) who had previously been treated in the Orthodontic Clinic at Sivas Cumhuriyet University Faculty of Dentistry. The patients were divided into two groups: Group 1: Myobrace (n = 18), and Group 2: twin block (n = 18). The effects of the appliances on the skeletal, dentoalveolar, soft tissue, craniocervical, and other anatomic structures were assessed using 46 measurements (22 linear and 24 angular), on pre and post-treatment cephalometric radiographs. AudaxCeph 5.0 software (Ljubljana, Slovenia) was used for the analysis. To analyze the changes after one year of treatment, a paired sample t-test and Wilcoxon signed-rank test were used. Intergroup comparison was performed using the Student t-test and the Mann–Whitney U test.

**Results:**

In the Myobrace and Twin-block groups, there was a significant increase in SNB (°) (*p* = 0.004, *p* = 0.001), IMPA (°) (*p* = 0.005, *p* = 0.001) and a significant drop in U1/SN (°) (*p* = 0.021, *p* = 0.005). The lengths of Cd–Gn (mm), Go–Pg (mm), and Cd–Go (mm) increased significantly in the Twin-block group (*p* = 0.003, *p* = 0.010, *p* = 0.001), whereas the Myobrace group did not change. Similarly, there was no significant difference in pharyngeal and soft palate measurements in the Myobrace group but a statistically significant decrease in SP length and angle in the Twin-block group (*p* = 0.001, *p* = 0.006). Increases in SN/OPT (°) (*p* = 0.032, *p* = 0.001) and SN/CVT (°) (*p* = 0.012, *p* = 0.001) were statistically significant in both groups. Myobrace was more difficult to use while sleeping, whereas the twin block caused more nausea.

**Conclusions:**

Both appliances can be used for mandibular advancement. The Twin-block appliance, on the other hand, was more effective and patient-friendly.

**Supplementary Information:**

The online version contains supplementary material available at 10.1186/s12903-023-02773-x.

## Background

Functional orthopedic appliances are used to manage mandibular retrognathia in skeletal Class II cases by stimulating condylar growth [1]. Since the first functional appliances were introduced by Robin in 1902 and Andresen in 1908, other clinicians have designed a wide range of new functional appliances [2–5].

Following conventional functional appliances such as the bionator and the activator, a new device called Pre-Orthodontic Trainer for Kids™ was developed in 1992 at the Myofunctional Research Center in Australia. It is now claimed that the trainer guides tooth eruption in the early mixed dentition, stimulates mandibular growth, and corrects abnormal myofunctional habits [6, 7]. In 2004, the same company launched the Myobrace System, which was developed as a different version of the first introduced trainer. This system has different appliance designs based on age groups and is available on the market in a variety of sizes. The Myobrace, unlike the first type of trainer appliance, facilitates dental arch development by applying light forces to the teeth via its Dynamicore structure. The structure is claimed to improve the arch shape and expand the dental arch [8].

Myofunctional appliances have been shown to improve the leveling of the lower dental arch and the inclination of the maxillary incisors [6, 7]. Furthermore, some researchers have reported that devices such as the T4K^®^ reduce overjet significantly in Class II division 1 cases [9, 10]. Several studies in the literature evaluate the dentoskeletal effects of the trainer appliance [9, 11, 12], but few assess the dentoskeletal effects of the Myobrace appliance [13].

Early orthodontic treatment of mandibular retrognathia has been reported to improve insufficient airway dimension in previous studies [14, 15]. Some researchers discovered that both Myobrace and Twin-block appliances have improved effects on airway dimensions [1, 16, 17].

According to some theory and research, breathing patterns impact head posture, which significantly influences craniofacial development [18]. According to Gresham and Smithells [19] and Morris et al. [20], children who do not have the habit of holding their head in an upright position are more likely to develop Angle Class II malocclusion, long face syndrome, and cervical spine kyphosis. Similarly, Sidlauskiene et al. [21] suggested a positive correlation between increased overjet and overbite and kyphotic posture. Regarding the Twin-block appliance's effects on cervical posture, there are conflicting findings in the literature. Some researchers reported no change in posture [22], while others asserted that it straightens the cervical posture [23]. To our knowledge, however, the current study is the first to evaluate the effect of Myobrace on cervical posture.

Because it does not articulate with other bones, the hyoid bone is referred to as a floating bone. Muscles and ligaments of the oropharyngeal complex attach it to the pharynx, skull, and mandible. The hyoid bone has three primary functions: swallowing, phonation, and breathing [24]. Mandibular position changes are known to affect the bone’s position and pharyngeal airway volume [25–27]. For instance, multiple studies showed that the Twin-block appliance advances the hyoid bone and widens the pharyngeal airway [28, 29]. However, no study has been found in the literature evaluating the effect of Myobrace on the position of the hyoid bone.

Functional appliances increase oropharyngeal dimensions by forcing the mandible, tongue, soft palate, and hyoid bone forward [30, 31]. Studies have shown that Twin-block treatment reduces the soft palate angle [1, 32]. However, the effect of Myobrace appliances on soft palate changes has not previously been researched.

Very limited clinical studies in the literature compare the skeletal, dental, soft tissue, and airway effects of these two appliances. On the other hand, clinicians are hesitant about using the Myobrace appliance, as its effectiveness is controversial, and there are some difficulties in its use.

The primary aim of this retrospective study was to compare the effects of the Twin-block and Myobrace appliances on skeletal, dental, and soft tissue and changes in the hyoid bone, cervical posture, soft palate, and airway. The secondary goal was to compare the appliances in terms of ease of use by assessing the factors that may influence patient compliance.

## Methods

### Participants

The current study involved thirty-six (19 females, 17 males; mean age, 12.14 ± 1.23) class 2 growing patients, who had been treated at the Orthodontic Department of Sivas Cumhuriyet University Dentistry Faculty. Written and verbal informed consents were separately obtained from the patients and their legal guards, and approval was obtained from the Clinical Research Ethics Committee of Sivas Cumhuriyet University (ID: 2020–02/05). This study was prepared per the Declaration of Helsinki. The sample size calculation using the G Power software revealed that at least 32 patients were required (effect size = 0.8, *α* = 0.05, and 1 − *β* = 0.90) [11].

The inclusion criteria were as follows: [12, 33] overjet > 4 mm, ANB > 4, class 2 molar and canine relationships, normal or decreased lower facial height, C3 cervical vertebral maturation stage, and no previous orthodontic treatment history. The exclusion criteria were as follows: post-pubertal growth period [34], lack of cooperation, facial syndromes, congenital anomalies, and temporomandibular joint diseases. The orthodontic archive provided information on 87 patients. Patients who did not meet the inclusion criteria (n = 21), had radiograph artifacts (n = 14), or had missing form data (n = 16) were excluded from the study. Data from 36 patients treated with twin blocks and Myobraces (18 in each group) who met the inclusion criteria were analyzed in this study.

The upper and lower parts of the Twin-block, whose effects were evaluated and routinely used in the orthodontic clinic, had labial bows and Adams clasps. Slow expansion screws were added to the upper part of the appliances in 16 patients with relative maxillary narrowness.

**Fig. 1:**
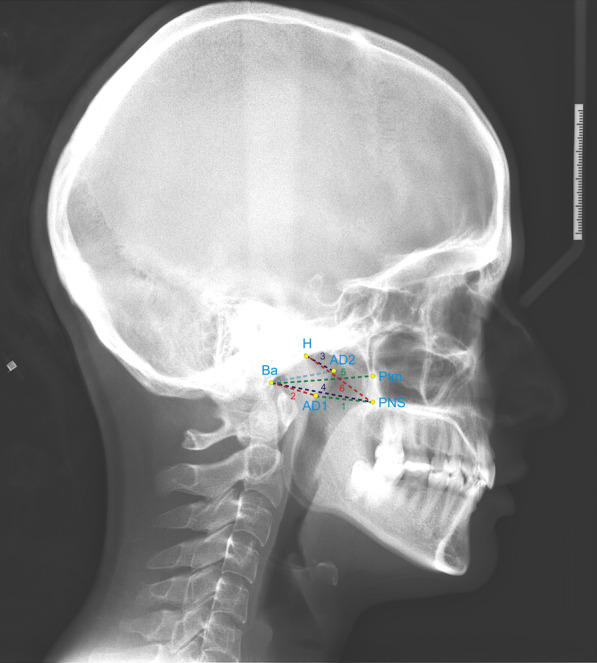
Pharyngeal airway linear measurements: **1** PNS–AD1 (mm), distance from PNS to AD1; **2** AD1–Ba (mm), distance from AD1 to Ba; **3** AD2–H (mm), distance between AD2 and H; **4** PNS–Ba (mm), distance from PNS to Ba; **5** Ptm–Ba (mm), distance from Ptm to Ba; **6** PNS–H (mm), distance between PNS and H

**Fig. 2:**
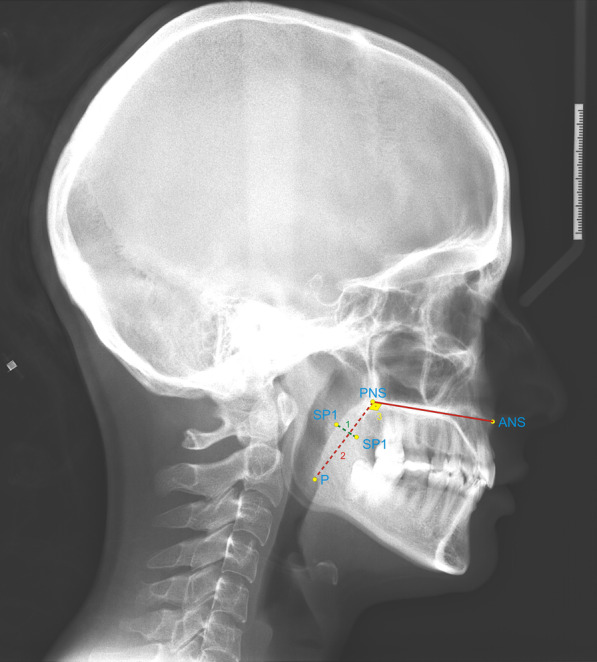
Soft palate measurements: **1** SP length, **2** SP thickness, and **3** SP angle. ANS, anterior nasal spine; PNS, posterior nasal spine, posterior limit of bony palate; P, lower tip of the uvula

**Fig. 3:**
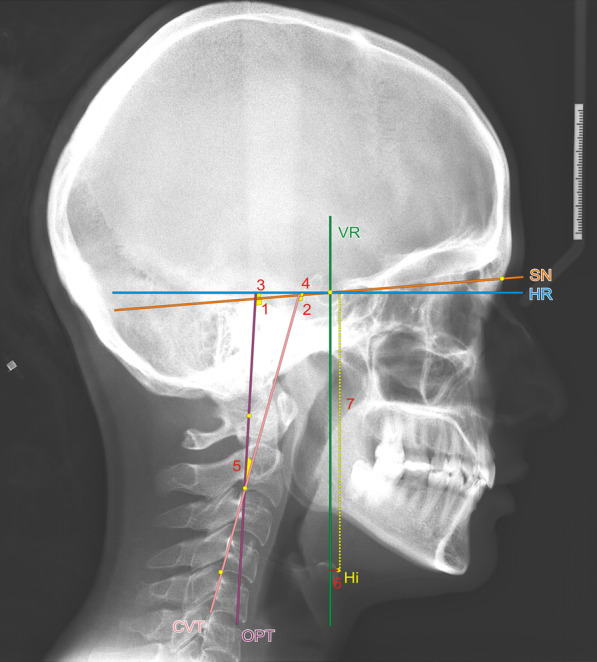
Craniocervical and hyoid measurements: CVT, cervical vertebra tangent: posterior tangent to the odontoid process through Cv4ip; OPT, odontoid process tangent: posterior tangent to the odontoid process through Cv2ip; HR, horizontal reference plane; VR, vertical reference plane; **1** SN-OPT (0), angle between SN and OPT; **2** SN-CVT (0), angle between SN and CVT; **3** OPT-HR (0), angle between OPT and HR; **4** CVT-HR (0), angle between CVT and HR; **5** OPT-CVT (0), angle between OPT and CVT; **6** Hi–VR (mm), sagittal distance from point Hi to VR; **7** Hi–HR (mm), vertical distance of Hi point to HR

Lateral cephalograms taken in the natural head position using the Orthoceph OC200D (Instrumentarium Dental, Tuusula, Finland) device were evaluated. AudaxCeph Version 5X software was used for cephalometric measurements (Ljubljana, Slovenia) (Table 1) [1, 12, 35], (Figs. 1, 2, and 3).

**Table 1:** Cephalometric measurements

Skeletal and dental measurements	
S	Sella, centre of sella turcica
N	Nasion, the most anterior point of the frontonasal suture in the midsagittal plane
A	Deepest midline point on the maxillary alveolar process’s anterior outer contour
B	Deepest point on the mandible’s anterior outer contour
U1	Long axis of the most anteriorly located upper central incisor
L1	Long axis of the most anteriorly located lower central incisor
Pg	Pogonion, point on the anterior surface of the chin that is the most forward-projecting
Go	Gonion, a point formed by the intersection of lines tangent to the ramus’s posterior border and the mandible’s lower border
Gn	Gnathion, the bony chin’s most anterior inferior point
Po	Porion, the central point on the upper margin of the external auditory meatus
Cd	Condylion, the most superior-posterior point of the mandibular condyle
Me	Menton, Lowest point on mandibular symphysis
Ar	Articulare, Intersection of the inferior surface of the cranial base and the posterior border of the ascending rami of the mandible
SNA (°)	Angle between Sella–Nasion and Nasion-A lines
SNB (°)	Angle between Sella–Nasion and Nasion-B lines
ANB (°)	Angle between Nasion-A and Nasion-B lines
U1–NA (mm)	Sagittal distance between the vestibule surface of the upper incisor and the Nasion-A plane
U1/NA (°)	Angle between the long axis of the upper incisor and the Nasion-A plane
L1–NB (mm)	Distance between the vestibule surface of the lower incisor and the Nasion-B plane in the sagittal direction
L1/NB (°)	Angle between the lower incisor’s long axis and the Nasion-B plane
Pg–NB (mm)	Distance between the Pogonion point and NB line
SN/GoGn (°)	Angle between the mandibular plane and the Sella–Nasion plane
Interinsizal açı	Angle between the long axis of the lower and upper central incisors
FMA (°)	Angle between Frankfort horizontal plane and mandibular plane
FMIA (°)	Angle between Frankfort horizontal plane and long axis of lower incisor
IMPA (°)	Angle between mandibular plane and long axis of lower central incisor
Cd–Gn (mm)	Distance between condylion and gnathion
Go–Pg (mm)	Distance between gonion and pogonion
Cd–Go (mm)	Distance between condylion and gonion
U1–SN (°)	Angle between long axis of upper incisor and Sella–Nasion plane
U1–PP (°)	Angle between long axis of upper incisor and palatal plane
FH/MP (°)	Angle between mandibular plane and Frankfort Horizontal plane
Overbite	Vertical distance between incisal edge of upper central incisor and incisal edge of lower central incisor
Overjet	Distance between vestibule surfaces of upper central incisor and vestibule surfaces of lower central incisor in sagittal direction
NAPg (°)	Angle between Nasion-A and A-Pogonion lines
SNPg (°)	Angle between Sella–Nasion and Nasion-Pogonion lines
MeGoArticulare	Angle between Gonion-Menton and Gonion-Articulare lines

Soft tissue measurements	
Gla-Sub-Pg (°)	Angle formed by the soft tissue Glabella, Subnasale, and Pogonion points
Nasolabial angle (Cl.Sn.SLS)	Angle formed by the Columella, Subnasale and Superior Labial Sulcus points
Mentolabial angle (Li.Ils.Pog)	Angle formed by Laberale inferius (Li)-inferior labial sulcus (Ils) and Ils-Pog’ points
Upper lip—E line	Distance from upper lip to Ricketts E plane
Lower lip E-line	Distance from lower lip to Ricketts E plane

Pharyngeal measurements	
PNS	Posterior nasal spine, posterior limit of bony palate
H	Hormion, point at the intersection of the skull base and the line perpendicular to S-Ba
AD2	Adenoid tissue along the line from PNS to H
AD1	Posterior pharyngeal wall along the line from PNS to Ba
Ba	Most inferior-posterior point on anterior margin of foramen magnum
Ptm	Most posterior point of pterygomaxillary fissure
PNS–AD1 (mm)	Distance from PNS to AD1
AD1–Ba (mm)	Distance from AD1 to Ba
AD2–H (mm)	Distance between AD2 and H
PNS–Ba (mm)	Distance from PNS to Ba
Ptm–Ba (mm)	Distance from Ptm to Ba
PNS–H (mm)	Distance between PNS and H

Soft palate measurements	
SP1	Anterior border of thickest part of uvula
SP2	Posterior border of thickest part of uvula
P	Lower end of uvula
SP thickness (mm)	Distance between SP1 and SP2 points
SP length (mm)	Distance between PNS and P points
SP angle (°)	Angle between ANS-PNS line and PNS-P line

Craniocervical and hyoid measurements	
CVT	Cervical vertebra tangent: posterior tangent to the odontoid process through Cv4ip
OPT	Odontoid process tangent: posterior tangent to the odontoid process through Cv2ip
HR	Horizontal reference plane
VR	Vertical reference plane
SN–OPT (°)	Angle between SN and OPT
SN–CVT (°)	Angle between SN and CVT
OPT–HR (°)	Angle between OPT and HR
CVT–HR (°)	Angle between CVT and HR
OPT–CVT (°)	Angle between OPT and CVT
Hi–VR (mm)	Sagittal distance from point Hi to VR
Hi–HR (mm)	Vertical distance of Hi point to HR

Patients undergoing functional treatment are routinely asked to complete a form called the ‘Pain and Discomfort Level Determination.’ The data on this form, which the patients filled out during the first week, first month, third month, and sixth month of treatment, were analyzed in the current study. Articles with similar topics were found by searching Google Scholar, Pubmed, and Web of Science databases with the keywords “dental pain and discomfort in functional appliances,” and eight items were created [33, 36]. The questions were sent to seven experts in the field. They rated each item as follows: (A) The item represents the feature, (B) It should be slightly corrected, (C) It should be highly corrected, and (D) The item does not represent the feature. The content validity study (CVI) was evaluated using the Davis technique (CVI = (A + B)/n, n = the total number of experts) [37]. In line with the experts’ suggestions, some items were corrected. CVI scores ranged from 0.9 to 1. It was accepted that all eight items had sufficient content validity because their CVI values were greater than 0.80 (Additional file 1) [38]. For the clarity of the questions, a pilot study was conducted with 10 patients, who were not included in the study.

A single investigator (H.C.) performed measurements without knowing which group each x-ray belonged to.

### Error of the method

The same investigator (H.C.) repeated measurements of ten randomly selected patients 15 days later. Repeated measurements were compared with the intra-correlation test. Correlation coefficient values for all measurements were greater than 0.762.

### Statistical analysis

SPSS statistical software (version 21.0; IBM Corp, Armonk, NY) was used for all analyses. The data homogeneity was evaluated with the Shapiro–Wilk test. Paired-sample t-test and the Wilcoxon signed-rank test were used for intra-group comparison of pre- and post-treatment data. The Student’s t-test and the Mann–Whitney U test were used for the inter-group comparison of data at T0 and T1. A *p* value < 0.05 was considered statistically significant.

## Results

The standard deviations and mean values of the T0 and T1 periods of the patients in both groups were calculated. Pre-treatment and post-treatment cephalometric values, intra-group, and inter-group comparisons were presented in Tables 2 and 3.

**Table 2:** Intragroup comparison of skeletal, dental, soft tissue, pharyngeal airway, cervical posture, hyoid bone position, and soft palate changes

	Myobrace T0–T1			Twin block T0–T1		
Parameters	Mean ± SD (T0)	Mean ± SD (T1)	*p*	Mean ± SD (T0)	Mean ± SD (T1)	*p*
SNA (°)	82.16 ± 2.66	82.36 ± 2.73	0.415	79.21 ± 3.03	78.77 ± 3.45	0.001*
SNB (°)	75.73 ± 2.67	76.34 ± 2.47	0.004*	73.95 ± 3.19	75.26 ± 3.56	0.001*
ANB (°)	6.40 ± 1.57	6.02 ± 1.81	0.178	5.26 ± 1.50	3.51 ± 1.61	0.001*
U1–NA (mm)	5.01 ± 1.49	3.83 ± 2.25	0.020*	5.03 ± 1.70	4.37 ± 5.97	0.050^a^*
U1/NA (°)	24.16 ± 8.14	19.17 ± 7.33	0.020*	23.36 ± 7.31	21.03 ± 5.43	0.003^a^*
L1–NB (mm)	5.51 ± 1.52	6.05 ± 1.53	0.026*	4.30 ± 1.88	5.90 ± 2.09	0.001*
L1/NB (°)	27.40 ± 5.64	30.47 ± 5.79	0.003*	23.77 ± 6.05	27.56 ± 4.46	0.001*
Pg–NB (mm)	2.48 ± 1.59	3.02 ± 2.54	0.146	2.29 ± 1.13	2.23 ± 1.19	0.001*
SN/GoGn (°)	29.95 ± 6.42	30.61 ± 6.58	0.429	31.48 ± 5.65	32.42 ± 6.14	0.001*
Interincisal Angle	122.03 ± 10.08	120.33 ± 9.33	0.279	127.59 ± 12.06	122.88 ± 7.47	0.010*
FMA (°)	22.59 ± 5.79	23.25 ± 7.14	0.360	24.26 ± 4.33	25.91 ± 5.02	0.001*
FMIA (°)	59.32 ± 5.30	55.82 ± 5.90	0.006*	60.61 ± 7.84	58.45 ± 6.09	0.005*
IMPA (°)	98.08 ± 7.81	100.92 ± 7.99	0.005^a^*	95.11 ± 6.79	97.64 ± 6.65	0.001*
Cd–Gn (mm)	103.33 ± 6.48	105.64 ± 10.98	0.248	100.23 ± 5.68	105.00 ± 7.13	0.003*
Go–Pg (mm)	65.00 ± 5.99	66.61 ± 7.57	0.188	63.47 ± 4.64	66.15 ± 4.13	0.010^a^*
Cd–Go (mm)	52.07 ± 5.16	53.50 ± 8.81	0.280	50.08 ± 5.27	53.01 ± 5.86	0.001*
U1/SN (°)	106.31 ± 8.04	101.43 ± 7.83	0.021*	102.58 ± 7.56	100.81 ± 6.39	0.005*
U1/PP (°)	114.48 ± 8.07	110.36 ± 7.36	0.059	111.48 ± 8.00	108.11 ± 6.50	0.157
FH/MP (°)	22.59 ± 5.79	23.25 ± 7.14	0.360	24.26 ± 4.33	23.91 ± 5.02	0.001*
Overbite	0.48 ± 0.46	0.38 ± 0.49	0.407^a^	0.72 ± 0.59	0.51 ± 0.56	0255^a^
Overjet	7.47 ± 1.46	4.55 ± 2.23	0.001*	6.94 ± 2.55	3.95 ± 1.75	0.216
NAPg (°)	169.26 ± 4.58	171.01 ± 6.32	0.042*	171.30 ± 4.12	175.01 ± 4.41	0.001*
SNPg (°)	77.15 ± 2.89	78.08 ± 3.24	0.003*	75.26 ± 3.21	76.51 ± 3.67	0.001*
MeGoArticulare	128.26 ± 6.19	127.60 ± 7.27	0.248	127.39 ± 5.84	126.18 ± 5.60	0.001*
Gla-Sub-Pg (°)	157.10 ± 6.69	159.76 ± 5.14	0.011^a^*	158.51 ± 5.40	162.85 ± 5.83	0.002*
Nasiolabial angle	113.67 ± 27.97	114.21 ± 9.22	0.717^a^	108.13 ± 10.48	110.61 ± 10.54	0.001*
Mentolabial angle	104.94 ± 15.35	113.30 ± 19.13	0.005*	111.07 ± 23.50	123.41 ± 19.43	0.003^a^*
Upper lip—E line	1.72 ± 2.15	1.89 ± 1.25	0.407^a^	1.98 ± 1.54	2.23 ± 1.78	0.731
Lower lip—E line	2.69 ± 2.59	1.90 ± 1.42	0.260^a^	1.74 ± 1.24	0.23 ± 1.60	0.382
PNS–AD2 (mm)	17.03 ± 44.13	21.54 ± 48.45	0.157^a^	20.09 ± 48.27	63.39 ± 146.92	0.485^a^
PNS–AD1 (mm)	22.48 ± 5.04	22.71 ± 4.66	0.671	22.51 ± 2.48	22.80 ± 3.01	0.776^a^
AD1–Ba	18.05 ± 3.32	18.65 ± 4.08	0.912	18.38 ± 2.32	17.73 ± 3.20	0.678
AD2–H (mm)	27.23 ± 4.39	27.80 ± 3.93	0.334^a^	26.23 ± 5.52	36.05 ± 27.65	0.069^a^
PNS–Ba (mm)	40.68 ± 5.19	40.80 ± 5.56	0.930	40.58 ± 3.44	40.78 ± 2.91	0.196^a^
Ptm–Ba (mm)	43.20 ± 4.49	42.88 ± 5.84	0.758	43.14 ± 3.18	41.83 ± 4.10	0.148^a^
PNS–H (mm)	44.16 ± 44.87	49.33 ± 50.48	0.872^a^	46.32 ± 51.84	99.45 ± 173.51	0.509
SP thickness (mm)	8.84 ± 1.33	9.14 ± 1.51	0.443	8.83 ± 1.31	9.63 ± 1.60	0.070^a^
SP length (mm)	28.77 ± 3.99	29.67 ± 4.71	0.122^a^	29.93 ± 3.16	29.35 ± 3.45	0.001*
SP angle (°)	131.90 ± 7.63	129.59 ± 7.48	0.153	133.30 ± 4.42	129.03 ± 5.77	0.006*
SN/OPT (°)	97.56 ± 11.15	100.81 ± 12.39	0.032*	102.45 ± 9.83	104.76 ± 9.98	0.001*
SN/CVT (°)	104.04 ± 10.73	107.73 ± 11.32	0.012*	106.78 ± 8.94	110.33 ± 8.92	0.001*
OPT/HR (°)	89.47 ± 7.27	86.03 ± 20.87	0.409^a^	93.62 ± 9.08	93.31 ± 9.18	0.986
CVT/HR (°)	95.95 ± 7.17	98.23 ± 9.94	0.192	97.981 ± 8.05	98.90 ± 8.56	0.001*
OPT/CVT (°)	6.48 ± 2.88	6.42 ± 3.31	0.317	15.60 ± 43.92	15.47 ± 3.27	0.036^a^*
Hi–VR (mm)	11.31 ± 7.06	14.54 ± 10.63	0.324^a^	8.05 ± 5.32	11.13 ± 7.18	0.105
Hi–HR (mm)	95.68 ± 7.22	97.62 ± 12.53	0.080^a^	93.12 ± 6.72	95.35 ± 8.20	0.001*

^a^Wilcoxon signed-rank test, *Statistically significant changes (*p* < 0.05)

SD: Standard deviation, Linear and angular cephalometric points measured: SNA, sella–nasion-point A angle; SNB, sella–nasion-point B angle; ANB, point A–nasion–point B angle; A/OLp, linear position of the maxillary base; Pg/OLp, linear position of the mandibular base; N-A-Pg, angle between points of nasion, A, and pogonion; SN/GoGn, the angle between Sella–nasion and gonion–gnathion planes; Co–A, maxillary length; Co–Gn, mandibular real length; L1–NB, lower incisor-nasion/point B line (mm and angle); IMPA, angle between lower incisor long axis and mandibular plane. 1/NA, upper incisor–nasion/point A line (angle and mm); U1/SN, angle between upper incisor long axis and sella–nasion plane; U1/L1, interincisal angle; Is/OLp, position of the maxillary central incisor; Ii/OLp, position of the mandibular central; Mi/OLp, position of the lower first molar; Ms/OLp, position of the upper first molar; Z angle, porion point/orbital point (Frankfort plane)—line E (Ricketts line profile) angle

**Table 3:** Intergroup comparison of skeletal, dental, soft tissue, pharyngeal airway, cervical posture, hyoid bone position, and soft palate changes

	Myobrace	Twinblock	
Parameters	Mean ± SD (T1–T0)	Mean ± SD (T1–T0)	*p* value
SNA (°)	0.20 ± 1.04	− 0.44 ± 0.97	0.554
SNB (°)	0.61 ± 0.80	1.31 ± 1.42	0.52^a^
ANB (°)	− 0.38 ± 1.19	− 1.75 ± 1.08	0.608
U1–NA (mm)	− 1.18 ± 2.0	− 0.66 ± 6.61	0.210
U1/NA (°)	− 4.99 ± 8.50	− 2.33 ± 6.60	0.560
L1–NB (mm)	0.54 ± 0.96	1.60 ± 1.29	0.074
L1/NB (°)	3.07 ± 3.83	3.79 ± 4.03	0.461
Pg–NB (mm)	0.54 ± 1.53	− 0.06 ± 0.60	0.257^a^
SN/GoGn (°)	0.66 ± 1.84	0.96 ± 1.44	0.409
Interincisal Angle	− 1.70 ± 8.98	− 4.71 ± 10.37	0.651
FMA (°)	0.66 ± 3.07	1.65 ± 2.95	0.703
FMIA (°)	− 3.50 ± 4.93	− 2.16 ± 4.15	0.730
IMPA (°)	2.84 ± 3.80	2.53 ± 4.42	0.617
Cd–Gn (mm)	2.31 ± 8.4	4.77 ± 4.60	0.310^a^
Go–Pg (mm)	1.61 ± 5.14	2.68 ± 3.70	0.861
Cd–Go (mm)	1.43 ± 5.59	2.93 ± 2.59	0.181^a^
U1/SN (°)	− 4.88 ± 8.89	− 1.77 ± 7.08	0.443
U1/PP (°)	− 4.12 ± 3.07	− 3.37 ± 2.95	0.703
FH/MP (°)	− 0.10 ± 0.44	− 0.35 ± 0.74	0.041*
Overbite	− 0.10 ± 2.27	− 0.21 ± 1.94	0.502
Overjet	− 2.92 ± 3.47	− 2.99 ± 1.93	0.331^a^
NAPg (°)	1.75 ± 1.15	3.71 ± 1.28	0.559^a^
SNPg (°)	0.93 ± 2.42	1.25 ± 2.31	0.648
MeGoArticulare	− 0.66 ± 5.06	− 1.21 ± 3.35	0.578
Gla-Sub-Pg (°)	2.66 ± 3.67	4.34 ± 7.50	0.086^a^
Nasiolabial angle	0.54 ± 11.48	2.48 ± 12.51	0.831
Mentolabial angle	8.36 ± 2.40	12.34 ± 1.63	0.450^a^
Upper lip—E line	0.17 ± 2.67	0.25 ± 1.40	0.354^a^
Lower lip—E line	− 0.79 ± 2.40	− 1.51 ± 1.54	0.055^a^
PNS–AD2 (mm)	4.51 ± 3.76	43.3 ± 3.22	0.475
PNS–AD1 (mm)	0.23 ± 3.87	0.29 ± 1.84	0.009*
AD1Ba	0.60 ± 6.03	− 0.65 ± 3.10	0.107^a^
AD2–H (mm)	0.57 ± 4.12	9.82 ± 3.16	0.572^a^
PNS–Ba (mm)	0.12 ± 4.39	0.20 ± 3.55	0.744
Ptm–Ba (mm)	− 0.32 ± 2.34	− 1.31 ± 1.82	0.055^a^
PNS–H (mm)	5.17 ± 1.66	53.13 ± 2.10	0.768^a^
SP thickness (mm)	0.30 ± 3.06	0.80 ± 2.60	0.765
SP length (mm)	0.90 ± 6.74	− 0.58 ± 5.65	0.192
SP angle (°)	− 2.31 ± 6.07	− 4.27 ± 6.20	0.943
SN/OPT (°)	3.25 ± 5.75	2.31 ± 6.25	0.855
SN/CVT (°)	3.69 ± 7.72	3.55 ± 6.8	0.980
OPT/HR (°)	− 3.44 ± 7.32	− 0.31 ± 6.65	0.976
CVT/HR (°)	2.28 ± 1.82	0.92 ± 4.58	0.038^a^*
OPT/CVT (°)	− 0.06 ± 10.26	− 0.13 ± 6.56	0.245^a^
Hi–VR (mm)	3.23 ± 8.91	3.08 ± 5.00	0.246^a^
Hi–HR (mm)	1.94 ± 7.2	2.23 ± 6.72	0.771

^a^Mann–Whitney U test, * Statistically significant changes (*p* < 0.05), SD: Standard deviation, Linear and angular cephalometric points measured: SNA, sella–nasion–point A angle; SNB, sella–nasion–point B angle; ANB, point A–nasion–point B angle; A/OLp, linear position of the maxillary base; Pg/OLp, linear position of the mandibular base; N-A-Pg, angle between points of nasion, A, and pogonion; SN/GoGn, the angle between Sella–nasion and gonion–gnathion planes; Co–A, maxillary length; Co–Gn, mandibular real length; L1–NB, lower incisor-nasion/point B line (mm and angle); IMPA, angle between lower incisor long axis and mandibular plane. 1/NA, upper incisor–nasion/point A line (angle and mm); U1/SN, angle between upper incisor long axis and sella–nasion plane; U1/L1, interincisal angle; Is/OLp, position of the maxillary central incisor; Ii/OLp, position of the mandibular central; Mi/OLp, position of the lower first molar; Ms/OLp, position of the upper first molar; Z angle, porion point/orbital point (Frankfort plane)—line E (Ricketts line profile) angle

### Skeletal and dental measurements

In the Myobrace group’s skeletal and dental measurements, there was a statistically significant increase in SNB (°), L1/NB (°) and L1–NB (mm), and IMPA (°) (*p* = 0.004, *p* = 0.003, *p* = 0.026), and a significant decrease in U1/NA (°) and U1–NA (mm) (*p* = 0.020, *p* = 0.020). U1/SN (°) and overjet showed statistically significant decreases (*p* = 0.021, *p* = 0.001) while NAPg (°) and SNPg (°) showed statistically significant increases (*p* = 0.042, *p* = 0.003).

In the Twin-block group, skeletal and dental measurements showed a statistically significant decrease for SNA (°) and ANB (°) (*p* = 0.001, *p* = 0.001), and a statistically significant increase for SNB (°) (*p* = 0.001). IMPA showed a statistically significant increase (*p* = 0.001). Increases in SN/GoGn (°) and FMA (°) were significant (*p* = 0.001, *p* = 0.001). A statistically significant decrease was observed in the interincisal angle (*p* = 0.010). The increase in the lengths of Cd–Gn (mm), Go–Pg (mm), and Cd–Go (mm) was statistically significant (*p* = 0.003, *p* = 0.010, *p* = 0.001).

### Soft tissue measurements

Gla-Sub-Pg (°) and labiomental angle increased statistically in the Myobrace group (*p* = 0.011, *p* = 0.005). The nasolabial angle, as well as the Gla-Sub-Pg (°) and mentolabial angles, increased significantly in the twin-block group (*p* = 0.001, *p* = 0.002, *p* = 0.003).

### Pharyngeal and soft palate measurements

In the Myobrace group, no statistically significant differences in pharyngeal and soft palate measurements were found (*p* > 0.05). In the Twin-block group, the decrease in SP length and SP angle was statistically significant (*p* = 0.001, *p* = 0.006).

### Craniocervical and hyoid measurements

In both Myobrace and Twin-block groups, the increases in SN/OPT (°) (*p* = 0.032, *p* = 0.001) and SN/CVT (°) (*p* = 0.012, *p* = 0.001) were statistically significant. CVT/HR (°) and Hi–HR (mm) in the Twin-block group showed statistically significant increases (*p* = 0.001, *p* = 0.001).

When the alterations that occurred in the T0–T1 interval were compared between groups, the changes in FH/MP (°) and PNS–AD1 (mm), and CVT/HR (°) measurements were found to be statistically significant (Table 4) (*p* = 0.041, *p* = 0.009, *p* = 0.038).

**Table 4:** Comparison of pain and discomfort levels

Sensation	T0 (1. Week)			T1 (1. Month)			T2 (3. month)			T3 (6. Month)		
	TB	MYO	*p* value	TB	MYO	*p* value	TB	MYO	*p* value	TB	MYO	*p* value
Nausea	1.56 ± 0.62	1.16 ± 0.30	0.001	1.38 ± 0.61	1.16 ± 0.37	0.014	1.31 ± 0.47	1.21 ± 0.41	0.194	1.38 ± 0.50	1.37 ± 0.59	0.660
Teeth sensitivity	2.06 ± 0.57	2.26 ± 0.65	0.159	1.69 ± 0.70	2.00 ± 0.57	0.052	1.69 ± 0.47	1.95 ± 0.62	0.805	1.81 ± 0.65	2.00 ± 0.66	0.581
Difficulty in using at sleep	1.25 ± 0.57	2.16 ± 0.76	0.134	1.25 ± 0.57	1.68 ± 0.82	0.019^*^	1.19 ± 0.40	1.58 ± 0.69	0.003^*^	1.19 ± 0.54	1.58 ± 0.60	0.059
Gum tissue pain and bleeding	1.56 ± 0.62	1.63 ± 0.59	0.711	1.31 ± 0.47	1.53 ± 0.61	0.083	1.06 ± 0.25	1.42 ± 0.50	0.001^*^	1.38 ± 0.61	1.32 ± 0.47	0.331
Pain	1.75 ± 0.57	2.21 ± 0.53	0.626	1.75 ± 0.68	2.11 ± 0.45	0.027^*^	1.63 ± 0.50	1.74 ± 0.56	0.966	1.75 ± 0.68	1.58 ± 0.50	0.382
Speech Impairment	2.19 ± 0.40	2.21 ± 0.71	0.020^*^	2.00 ± 0.51	2.26 ± 0.65	0.035^*^	2.00 ± 0.36	2.11 ± 0.56	0.061	2.13 ± 0.61	1.95 ± 0.62	0.801
Saliva increment	1.94 ± 0.77	2.11 ± 0.87	0.272	1.94 ± 0.85	2.00 ± 0.74	0.290	1.81 ± 0.65	1.79 ± 0.71	0.581	1.75 ± 0.57	1.95 ± 0.70	0.834
TMJ pain	1.31 ± 0.60	1.37 ± 0.49	0.970	1.25 ± 0.44	1.26 ± 0.45	0.864	1.25 ± 0.57	1.42 ± 0.69	0.193	1.44 ± 0.51	1.26 ± 0.45	0.070

*MYO* Myobrace, *TB* Twin block. Student t-test results, *p < 0.05

### Findings of the questionnaire

The 1st week, 1st month, 3rd month, and 6th-month survey data mean, standard deviations, and intergroup comparison results of the groups were shown in Table 4.

In the first week, the groups significantly differed in terms of speech disorder and nausea (*p* = 0.020, *p* = 0.001). When the first-month data were analyzed in terms of nausea, difficulty using while sleeping, pain, and speech impairments, the difference between the groups was statistically significant (*p* = 0.014, *p* = 0.019, *p* = 0.027, *p* = 0.035). Variations such as use in sleep, appliance discomfort, and gingival bleeding significantly differed in the third month (*p* = 0.003, *p* = 0.001).

## Discussion

The present study was quite comprehensive as it simultaneously evaluated skeletal, dental, soft tissue, pharyngeal airway, soft palate, craniocervical posture, and hyoid bone effects of the Twin-block and Myobrace appliances. Additionally, the two appliances were compared in terms of ease of use. To the best of our knowledge, no comprehensive research on the effects of these two appliances has ever been conducted.

At the end of the treatment, both groups showed statistically significant changes in skeletal and dental measurements, but the Twin-block treatment appeared to be more effective in several categories [11, 39]. In the Twin-block group, the increase in SNB (°), decrease in ANB (°), and changes in mandibular length were more noticeable. Ghodke et al. [16] found comparable results in terms of Twin-block effectiveness. In the current study, the Myobrace group revealed no significant change in mandibular length, similar to the findings of Idris et al. [12] and Usumez et al. [9]. There was a significant increase in the lower incisor angle in both groups, consistent with the study results of Elhamouly et al. [11] However, several studies found that using a trainer or activator had little effect on the angle of the lower incisors [12]. In the current study, the protrusion of the lower incisors in both groups may be related to the mechanism of action of the appliances. The lower incisors may protrude by hitting the acrylic in the Twin-block appliance while the muscles are trying to return the mandible to its original position. Significant proclination can be seen in the lower incisors with the lip bumper effect of Myobrace. [11].

Twin block was a more effective mandibular advancement technique compared to Myobrace. The positive skeletal changes obtained with the Twin-block appliance may be because it is a custom-made device that allows precise anterior mandibular positioning. Furthermore, the twin block is made of acrylic, which is harder than Myobrace material. While previous research showed the ability of Myobrace in mandibular advancement to be limited, the current study found that it plays an active role in mandibular advancement. Generally, the Myobrace appliance is made of a more flexible material than the twin block is, making it difficult for patients to keep their mandibles in a forward position [12]. The medium hard form of the Myobrace was used in the current study to make it easier for patients to hold their mandible forward.

In contrast to the Myobrace group, the significant increase in vertical growth angles observed in the Twin-block group could be attributed to the bite-opening effect of the Twin-block appliance and the guided extrusion of the posterior teeth [40, 41]. Unlike previous studies [10, 12], soft tissue measurements of the Myobrace showed a significant improvement (Gla-Sub-Pg and mentolabial angle). The improvement in the Twin-block group, on the other hand, was greater than in the Myobrace group.

The widening of the nasopharyngeal and hypopharyngeal regions after mandibular advancement using orthopedic appliances indicates that the airway is affected by the mandibular position [9]. Pavoni et al. [42] found that using a conventional functional appliance for mandibular advancement increased the dimensions of the pharyngeal airway, PNS–AD1 and PNS–AD2. However, in this study, they observed a significant decrease in upper adenoid dimensions (AD2–H). Myobrace has been shown to have similar airway-widening effects by some research [17]. In the current study, both groups showed a statistically insignificant increase in airway measurements. The authors of the current study thought that by improving tongue position, the anterior movement of the mandible and the protrusion of the incisors in both groups may have increased the volume of the airway. The severity of the malocclusion, variations in ages, treatment times, and appliance types are just a few of the variables that may have contributed to the findings of the current study differing from those of earlier studies.

Chewing, breathing, and phonation are all influenced by the relationships between the soft palate and pharyngeal airway diameters. Ghodke et al. [16] observed that the SP angle in the Twin-block group decreased significantly, which is similar to the findings of the current investigation. Jena et al. [43] reported that the SP length and angle decreased significantly in both groups in their study comparing two different mandibular advancement devices, whereas SP thickness increased in the Twin-block group. In the current study, a significant change in soft palate measurements was observed in the twin-bock group, but not in the Myobrace group. Ghodke et al. [16] compared the Twin-block group with the control group, unlike the current study, and examined the 6-month changes. In their 6-month follow-up study, Jena et al. [43] compared the class I control group, class II control group, Mandibular Protraction Appliance-IV group, and Twin-block group.

The hyoid bone’s position is clinically significant as it is essential for maintaining upper airway dimensions. Rizk et al. [44] reported that mandibular advancement causes the anterior movement of the hyoid bone. Bavbek et al. [31] observed that after Forsus treatment, the hyoid bone moved forward significantly with no significant change in the vertical direction. The hyoid bone moved significantly in the sagittal direction in the Twin-block group in the current study without significant change in the vertical direction. In the Myobrace group, no significant movement was observed in the hyoid bone in the sagittal and vertical directions. As the Twin-block group’s mandibular advancement was greater than that of the Myobrace group’s, the Twin-block group’s sagittal change in the hyoid bone position is expected to be greater. Ozdemir et al. [32] found no change in hyoid bone position after fixed functional treatment of class II malocclusion. The fact that the patients were in the post-peak growth period may have contributed to the fact that the location of the hyoid bone in the study did not change.

In the current study, a statistically significant increase was observed in the SN/OPT and SN/CVT angles in both Twin-block and Myobrace groups. In other words, both treatment methods resulted in a more upright craniocervical posture. The skeletal system’s physiological growth processes and the interactions between bones and muscles affect cervical posture. The cervical spine and the mandible are anatomically related to one another [45]. Besides, the cervical spine is related to mandible size and craniofacial morphology [46]. According to Alsheikho et al. [22], functional orthopedic treatment did not affect craniocervical posture. The Bionator appliance was also used in this study conducted on Syrian patients. Kamal et al. [23] also suggested that functional treatment did not affect craniocervical angles. Significant changes in the CVT/HR and OPT/CVT angles were detected in the Twin-block group in the current study, which is consistent with other cervical vertebral changes. Similarly, Aglarci [47] observed a significant change in mid-cervical posture (OPT–CVT (°)) with the use of twin blocks.

The ease of use of orthodontic appliances is critical to patient compliance. Nausea was detected at a higher rate in the Twin-block group than in the Myobrace group during the early stages of treatment (1st week and 1st month). This could be because the Twin-block appliance covers a larger area of the palate. In the first and third months of treatment, patients in the Myobrace group had significantly more difficulty using it while sleeping than patients in the Twin-block group. In terms of retention, the customized produced twin block was already expected to outperform the Myobrace.

The pain was found to be higher in the Myobrace group in the first month of the current study. Idris et al. found moderate pain in both the trainer and the activator groups during the first months of treatment, but the pain was greater in the activator group [33]. In this study, the medium hard form of the Myobrace appliance was used, which could explain the increased level of pain.

Speech difficulty was significantly higher in the Myobrace appliance than in the Twin-block appliance. Similarly, in a previous study, fewer speech difficulties were reported in the Twin-block group [33]. The Myobrace contains a double barrier (lingual and buccal oral screens) and additionally includes a tongue tag and tongue guard. The current study’s authors suggest that the structure of the Myobrace causes speech difficulty. The groups did not significantly differ in terms of temporomandibular joint pain.

### Limitations

In the current study, 3D structures were evaluated using 2D cephalometric X-rays. Three-dimensional image evaluations generate more reliable results. However, in terms of radiation dose, the use of 3D imaging techniques in children is debatable. Because of its low cost and low radiation dose, cephalometric X-ray is more convenient.

The results of Myobrace and Twin-block appliances after a year were analyzed in the current study. Another limitation of the study includes not knowing the long-term effects of the appliances and not being able to assess the possibility of relapse.

## Conclusions

- Both appliances can be used for mandibular advancement. However, the Twin-block appliance was more effective.
- The Twin-block appliance was convenient to use and more widely accepted by patients.
- As the long-term effects of Myobrace on mandibular advancement are unknown, the findings of the current study should not be generalized.

## Supplementary Information

**Additional file 1. Supplementary Table**: Pain and discomfort level determination questionnaire.

## Data Availability

The datasets created and/or analyzed during the current study are not publicly available due to [Ethics committee decision], but are available from the corresponding author upon reasonable request.
