# Supplementary material for: Dentoalveolar, skeletal, pharyngeal airway, cervical posture, hyoid bone position, and soft palate changes with Myobrace and Twin-block: a retrospective study
Source: BMC Oral Health. 2023 Jan 30;23:53. doi: 10.1186/s12903-023-02773-x (PMC9887833; doi:10.1186/s12903-023-02773-x)
Supplement: Supplementary file 1 — Additional file 1. Supplementary Table: Pain and discomfort level determination questionnaire. [file 12903_2023_2773_MOESM1_ESM.docx]

| Dear Friends,  Here are some sentences organized into groups. Take your time reading each sentence. Our goal is to learn how you feel while wearing the appliance we provide. Mark with an (X) the situation that best suits you (not at all, a little, a lot). |
| --- |
| 1. Do you experience nausea when you have the appliance in your mouth?  Not at all( ) A little( ) A lot( ) |
| 2. Do you experience tooth sensitivity while wearing the appliance?  Absolutely not( ) A little bit( ) A lot( ) |
| 3. Do you find it difficult to use the appliance while sleeping?  Absolutely not( ) A little bit( ) A lot( ) |
| 4. Have you experienced gum pain or bleeding since using the appliance?  Not at all( ) A little( ) A lot ( ) |
| 5. Do you have pain in your teeth?  Not at all( ) Somewhat( ) A lot( ) |
| 6. Do you find it difficult to speak with the appliance in your mouth?  Not at all( ) A little( ) A lot( ) |
| 7. Has the amount of saliva increased since you started using the appliance?  Absolutely not( ) A little bit( ) A lot( ) |
| 8. Have you experienced joint pain since you began using the appliance?  Not at all( ) A little( ) A lot( ) |

**Supplementary Table: Pain and discomfort level determination questionnaire**
